# Large-Scale ALS Data Semantic Classification Integrating Location-Context-Semantics Cues by Higher-Order CRF

**DOI:** 10.3390/s20061700

**Published:** 2020-03-18

**Authors:** Wei Han, Ruisheng Wang, Daqing Huang, Cheng Xu

**Affiliations:** 1 College of Electronic and Information Engineering, No. 29 Yudao Road, Nanjing University of Aeronautics & Astronautics, Nanjing 210016, China; 2 Department of Geomatics Engineering, University of Calgary, 2500 University Drive NW, Calgary, AB T2N 1N4, Canada

**Keywords:** airborne laser scanning, semantic classification, feature extraction, conditional random fields, higher-order potentials

## Abstract

We designed a location-context-semantics-based conditional random field (LCS-CRF) framework for the semantic classification of airborne laser scanning (ALS) point clouds. For ALS datasets of high spatial resolution but with severe noise pollutions, more contexture and semantics cues, besides location information, can be exploited to surmount the decrease of discrimination of features for classification. This paper mainly focuses on the semantic classification of ALS data using mixed location-context-semantics cues, which are integrated into a higher-order CRF framework by modeling the probabilistic potentials. The location cues modeled by the unary potentials can provide basic information for discriminating the various classes. The pairwise potentials consider the spatial contextual information by establishing the neighboring interactions between points to favor spatial smoothing. The semantics cues are explicitly encoded in the higher-order potentials. The higher-order potential operates at the clusters level with similar geometric and radiometric properties, guaranteeing the classification accuracy based on semantic rules. To demonstrate the performance of our approach, two standard benchmark datasets were utilized. Experiments show that our method achieves superior classification results with an overall accuracy of 83.1% on the Vaihingen Dataset and an overall accuracy of 94.3% on the Graphics and Media Lab (GML) Dataset A compared with other classification algorithms in the literature.

## 1. Introduction

The semantic classification has been, and still is, of significant interest to the Light Detection and Ranging (LiDAR) processing and machine learning. Airborne laser scanning (ALS) system can acquire both geometric and radiometric information of geo-objects, which has been widely used in semantic classification [1]. An increasing number of applications require the result of semantic classification ranging from object detection to automatic three-dimensional (3D) modeling. Automated urban object extraction from remotely sensed data, especially from ALS point clouds, is a very challenging task due to the complex urban environments and the unorganized point clouds data. We also consider finding different types of objects in a small local neighborhood in this paper, which is obviously difficult for reliable extractions. Compared with a binary decision process, each 3D point in the irregularly distributed point clouds is assigned with a semantic object label in this work. However, due to the obvious defects of ALS point clouds (e.g., noise, inhomogeneity, loss of sharp features and outliers), current methods are not resilient for clutter scenes and heterogeneous ALS point cloud data obtained from different equipment. Therefore, we incorporate location, spatial contextual, and semantics cues within a higher-order conditional random field (CRF) framework to provide complementary information from varying perspectives, so that it can address the common misjudgment of semantic classes in ALS point clouds, from the perspectives of the accuracy of each class and the overall accuracy.

### 1.1. Related Works for ALS Point Cloud Classification 

According to the type of entity used for classification, existing methods can be categorized as point-based and cluster-based (or segment-based) [2,3]. Point-based methods classify each point of the ALS data by using features as the inputs for supervised or unsupervised classifiers [4], while cluster-based methods segment the ALS data into clusters, then class labels are assigned to the clusters in which all points share the same class label [5,6]. We briefly review the aforementioned methods, and demonstrate the rationale for our method in what follows.

Point-based methods generally extract point-wise features locally from the neighborhood defined by a sphere or cylinder. Therefore, such methods usually focus on the selection of discriminative features and effective classifiers. For instance, Reference [7] worked on 3D scene analysis, including geometric features extraction and optimal neighbors selection. Then an optimal eigenentropy-based scale selection method was proposed. Reference [2] combined airborne LiDAR with images to extract more discriminative features. Then, based on these features, the points were classified with AdaBoost. Spectral information from full waveform ALS point clouds can also be used for feature extraction, which exhibit promising results in large-scale urban environments [8]. To take advantage of contextual information, Reference [9] integrated a random forest (RF) classifier into a CRF framework, where the unary and pairwise potentials of CRF were based on probabilities computed by RF. With multispectral images and 3D geometry data, fully connected conditional random field (FCCRF) graph model was employed [10]. Although these point-based methods benefit from using contextual information, their effects have been very limited because they merely consider the coherences between points within a small neighborhood or employ the FCCRF model with great computational cost. Moreover, point-based features are sensitive to noise, such that these methods may not be suitable for complicated scenes and noisy ALS cloud points. 

Cluster-based methods choose a point segment in which points share homogeneous properties as the entity to be classified. A variety of methods has been used, which can be roughly classified as exclusive methods, overlapping methods, hierarchical methods and probabilistic methods [11,12]. It has also been proposed to use different entities in the form of voxels, blocks and pillars [13], in the form of planes, smooth surfaces and rough surfaces [6], or in the form of spatial bins, planar segments and local neighborhoods [14]. A robust unsupervised clustering algorithm P2C based on a hierarchical analysis was proposed by Reference [15], which comprises two stages: segmenting the point cloud into non-overlapping patches, and merging the patches into surfaces according to their probability density functions (PDFs). Reference [16] proposed a probability density clustering algorithm to perform hierarchical clustering and a higher-order CRF model was employed based on two different clusters. A natural exponential function was used to obtain hierarchical clusters of ALS point cloud [17], then multilevel point cluster-based features were extracted with latent Dirichlet allocation and sparse coding. Point-homogeneity constraint clustered points with similar geometric and radiometric properties in Reference [18], and markov random field (MRF) model was built based on these clusters. Recently, [19] introduced a super-point graph (SPG) to segment large-scale point clouds. Cluster-based methods uses more semantic features, like cluster size and shape, and therefore obtains a smoother classification result than the point-based method. However, the performance of cluster-based method is negatively affected by under/over-segmentation errors and loss of information. The semantic classification results are strongly connected to the accuracy of cluster-based method.

Specifically, the launch of deep learning in point cloud classification causes significantly promising classification results. PointNet [20] and following works Point-Net++ [21], Recurrent Slice Network (RSNet) [22], Dynamic Graph Convolutional Neural Networks (DGCNN) [23], and Point Convolutional Neural Networks (PointCNN) [24] further focus on exploring the local context and hierarchical learning architectures. [1] transformed the 3D neighborhood features of a point into 2D images, which were treated as the input of a multi-scale convolutional neural networks (CNN) for training and testing tasks. However, most of the above methods are used in indoor point clouds. Due to the attributes of ALS point cloud, which is generally coarse, noisy, sparse and heterogeneous, the process of model training may be time-consuming and the model may not be suitable for ALS point clouds from different equipment. Then, the present study describes a combination of point-base and cluster-based method in a CRF framework, which takes full advantages of location, context and semantics cues and shows high plausibility in ALS point cloud semantic classification compared with others.

### 1.2. Contribution 

In this paper, an efficient algorithm for the ALS point cloud semantic classification is proposed to improve the overall accuracy and accuracy of each class, especially for classes with small size, which is rarely studied in other papers. Higher-order potentials of the location-context-semantics-based conditional random field (LCS-CRF) play an important role in semantic classification. To increase the efficiency, the Cloth Simulation Filter (CSF) is analyzed and further used to remove ground points with RANdom SAmple Consensus (RANSAC) algorithm for the cluster-based features extraction. Then, a constrained mean-shift clustering method is proposed to obtain clusters which are used to define semantic rules. The higher-order potential and unary potential can be fused based on the class membership probabilities for the inference of LCS-CRF algorithm. The efficiency of the proposed LCS-CRF method is confirmed with two ALS datasets. Compared to the other semantic classification methods, the experimental results confirm that the LCS-CRF algorithm shows a qualitative performance.

The rest of this paper is organized as follows: In Section 2, details of the proposed ALS point cloud semantic classification method are elaborated. Section 3 presents the experimental results produced by the proposed algorithm, and the paper concludes with Section 4.

## 2. Methodology

**Figure 1:**
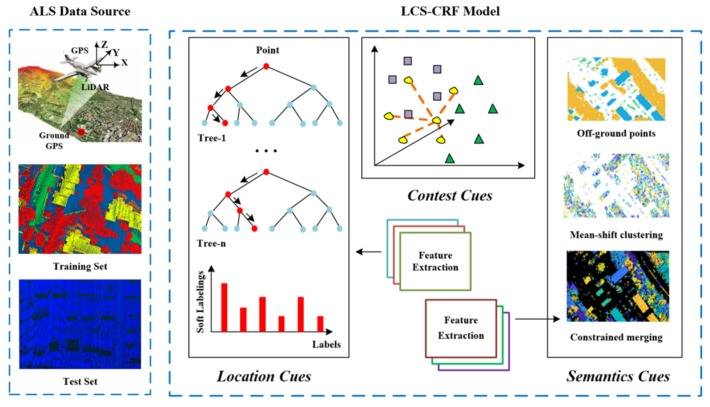
Flowchart of the location-context-semantics-based conditional random field (LCS-CRF) algorithm integrating location, context, and semantics cues. ALS = airborne laser scanning.

It is the goal of this paper to present an efficient CRF-based framework for semantic classification from ALS point cloud data without the use of image data providing spectral information. Firstly, multiple features of ALS point clouds are processed mainly based on their locations which can efficiently improve the results of the point-based classification process. Secondly, a Random Forests (RF) classifier is employed to produce the soft labeling results. Some outliers are found in the initial semantic result, then, a CRF framework is presented to smooth the result with context information between neighboring points. However, we find that it is of low accuracy for the objects with a small size, especially for cars. LCS-CRF is proposed to solve this problem and can achieve higher overall accuracy with a higher-order potential. Cluster-based features are extracted on the cluster obtained by a constrained mean-shift clustering method and semantic rules are defined. Then, based on the common knowledge of semantic rules, we define the higher-order potentials. Finally, the location, context and semantics cues are, respectively, encoded by unary, pairwise and higher-order potentials. Once fused, they can provide complementary information from varying perspectives, to improve the ALS point cloud semantic classification performance. A mean-field approximate inference method is employed to obtain the semantic classification results. Figure 1 shows the flowchart of the proposed method.

### 2.1. Feature Extraction 

#### 2.1.1. Point-Based Feature Extraction

Three types of features are employed in this section, geometric features from the ALS point cloud properties, local shape features from the structure tensor and primitive features from the data source. Since the distinctiveness of point-based features strongly depends on the respective neighborhood encapsulating those 3D points, a data-driven approach is proposed to determine the neighborhood size by selecting the number of nearest neighbors in the local 3D neighborhood of each individual point with eigenentropy-based scale selection [7]. The neighbor size can be determined based on the minimum eigenentropy by varying values of the scale parameter:

(1) $$E_{i,\lambda }(k)=-\sum_{s=1}^{3}\lambda_{i,s}(k)\ln\lambda_{i,s}(k),$$

(2) $$k_{i}^{*}=\underset{k\in \kappa }{\arg}\;\min E_{i,\lambda }(k),$$

where $E_{i,\lambda }(k)$ is the eigenentropy of *i*th point based on the scale parameter k, and $k_{i}^{*}$ represents the optimal value for *i*th point. Three eigenvalues ($\lambda_{s}$, s = 1, 2, 3) can be derived by the symmetric positive semi-definite 3D structure tensor $T\in {\mathbb{R}}^{3\times 3}$, which is obtained by the k nearest neighbors of each point. In the scope of our work, scales parameters within an interval $\kappa =[k_{\min},k_{\max}]$ are considered, with a lower boundary of $k_{\min}$ = 10 neighbors to remain robustness statistically [25,26,27] and an upper boundary of $k_{\max}$ = 100 to limit the computational effort.

After the recovery of local neighborhoods, we congregate some features which well-suit this semantic classification for ALS point cloud. The features used in our work are shown in Table 1. The point-based feature vector comprises 34 elements. 

Height H above Digital Terrain Model (DTM) is a discriminating feature to distinguish different classes. The DTM can be generated based on the local topography of the scene [26]. General geometric properties are represented by the radius r of the sphere encompassing k nearest neighbors and the maximum difference ΔH within the neighborhood. Density D, principle curvatures k1 and k2, Gaussian curvature $C_{g}$, mean curvature $C_{m}$, and verticality V [28] are used to describe the basic properties of ALS data, which has been demonstrated their efficiency by feature important analysis. Normal vector relationships N and curvature C (i.e., normal change rate) are also derived in this work. σ(⋅) means the variance of above geometric features in a sphere of radius r. With the k nearest neighbors of each point, 3D structure tensor $T\in {\mathbb{R}}^{3\times 3}$ can be derived to obtain 8 local shape features: linearity L, planarity P, scattering S, omnivariance O, anisotropy A, eigenentropy E, sum of eigenvalues $\sum_{Es}$, and change of curvature ΔC. Intensity I obtained directly by the ALS laser and its variance σ(I) in a sphere of radius r comprise the primitive feature set. In analogy to the 3D case, 2D projection of the 3D points onto XY-plane can reveal complementary information, especially for perfectly vertical structures. Then, $r_{2d}$ defined by the circle encompassing k nearest neighbors, 2D structure tensor $T\in {\mathbb{R}}^{2\times 2}$ (sum of eigenvalues $\sum_{Es,2d}$, ratio of eigenvalues $R_{2d}$), density $D_{2d}$ [29], and its variance $\sigma (D_{2d})$ are also derived as the elements of the point-based feature vector. 

#### 2.1.2. CSF with RANSAC

To increase the efficiency of LCS-CRF, off-ground points are employed to extract cluster-based features for the higher-order potentials. CSF [30] algorithm can be used to extract off-ground point from LiDAR data, which has been shown superior performance compared with other ground filtering methods. 

Two difficulties should be overcome for the ground filtering for ALS point cloud, i.e., insufficient information of small size objects for clustering which will have an obvious effect on the class accuracy, overall accuracy of classification result [17], and misjudgment between ground and classes with lower height (e.g., low vegetation). Then, RANSAC [31] is integrated with CSF to solve these problems, which is able to segment ground and off-ground points simultaneously. Pseudocode of Algorithm 1 for the RANSAC-based CSF algorithm is shown in Appendix A. 

**Figure 2:**
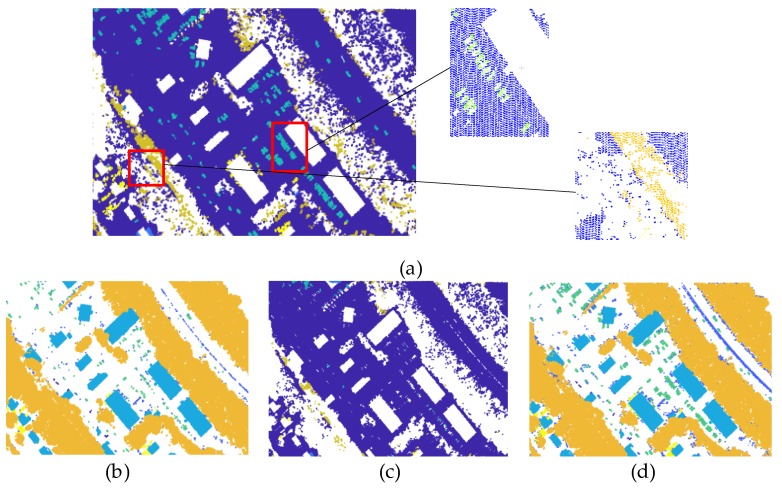
Results contrast between Cloth Simulation Filter (CSF) and Algorithm 1: (**a**) Ground points obtained from CSF algorithm. Details of misjudgment are also shown; (**b**) off-ground points obtained from CSF algorithm. Much information of small size objects is lost; (**c**) ground points obtained from Algorithm 1. Error samples between ground and classes are obviously refined; (**d**) off-ground points obtained from Algorithm 1. Enough information of small size objects, which directly affects the results, can be provided by higher-order potential.

Off-ground points set is generated in Algorithm 1 and the result is shown in Figure 2. More information of small size objects (e.g., car) and lower error samples between ground and classes are obtained. Then, clustering is performed on the off-ground points.

#### 2.1.3. Off-Ground Points Clustering

In this section, we first derive an over-segmentation of ALS point cloud by applying the mean-shift algorithm [32,33], a mountain climbing algorithm based on kernel density estimation without the need to initially specify the number of clusters. An adaptive gradient ascent is applied in the iterations of this algorithm, where shift vector value ‖m‖ will be larger in areas of low point density and lower in areas of high point density [4]. An isotropic Gaussian kernel Γ is adopted, and shift vector value ‖m‖ of point x can be defined as:

(3) $$\left\|m(x)\right\|=\left\|\frac{\sum_{i\in S_{r}}x_{i}\Gamma \left(\frac{{\left\|x-x_{i}\right\|}^{2}}{\gamma }\right)}{\sum_{i\in S_{r}}\Gamma \left(\frac{{\left\|x-x_{i}\right\|}^{2}}{\gamma }\right)}-x\right\|,$$

where $S_{r}$ represents the set of current point’s neighbors within the radius of r, and γ denotes the kernel width selected based on the point distribution for a considered scene. 

In this work, off-ground ALS data is heterogeneous and it is hard to distinguish different classes closed to each other in distance space (e.g., car and building or building and vegetation). Then, a constrained mean-shift algorithm is proposed, i.e., a post-processing step for the initial over-segmentation performed by mean-shift algorithm and two initial clusters with a low dissimilarity are preferred to be combined into one cluster. Two constraints are used for the dissimilarity discriminate between initial clusters:

- Constraint 1: local connectivity
  Local connectivity can be measured by the minimum Euclidean distance between points $p_{1}$ and $p_{2}$ obtained by
  (4) $$d(c_{m},c_{n})\leq th_{d}\;s.t.\;(c_{m},c_{n})=\arg\underset{\left(p_{1},p_{2}\right)}{\min}d(p_{1},p_{2}):p_{1}\in c_{m},p_{2}\in c_{n},$$
  where d(⋅) is the Euclidean distance between initial cluster $c_{m}$ and $c_{n}$, and $th_{d}$ is the threshold of the constraint.
- Constraint 2: structure correlation
  (5) $$\left|\right|\log T_{m}-\log T_{n}|{|}_{F}\leq th_{t},$$
  where $T_{m}\in {\mathbb{R}}^{3\times 3}$ and $T_{n}\in {\mathbb{R}}^{3\times 3}$ are 3D tensor structures for m th and n th clusters, log(⋅) the matrix logarithm operator, $||\cdot |{|}_{F}$ the Frobenius norm [34], and $th_{t}$ the threshold of the constraint.

The pseudocode of Algorithm 2, which shows the details of the constrained mean shift algorithm, is presented in Appendix B. 

**Figure 3:**
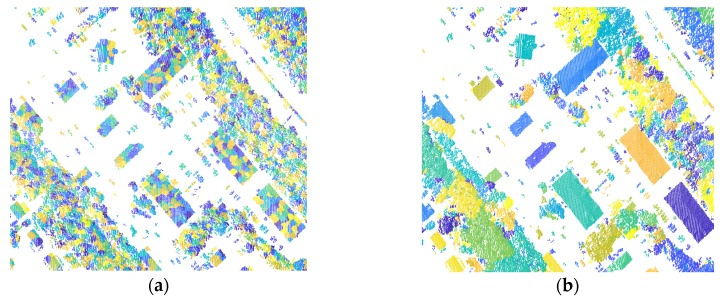
Results contrast between mean-shift algorithm and Algorithm 2: (**a**) Initial clusters achieved with mean-shift algorithm; (**b**) final clusters obtained by a post-processing step with constrained mean-shift algorithm.

Clusters of different classes exhibit different characteristics, which can be used to extract more discriminative cluster-based features. Clusters derived from mean-shift algorithm, as shown in Figure 3a, are scattered and cluttered, which cannot show the special information for different classes. But, as shown in Figure 3b, more accuracy and discriminative information are provided to perform cluster-based feature extraction.

#### 2.1.4. Cluster-Based Feature Extraction

In contrast to point-based feature extraction, features of cluster are extracted in this section. Point-based features can describe the details of a single point, whereas whole level information for different classes can be obtained from clusters and used to derive higher-order potentials. Herein, five features are extracted from each cluster:

- Hight $F_{H}$
  Hight above ground measured by the barycenter of the cluster is used to distinguish the roofs and other classes (e.g., cars, low vegetations), as even the lowest roofs are generally higher than cars or low vegetations.
- Distribution of ground points $F_{G}$
  A circular region centered on the cluster center can be divided in to angular bins. The distribution of ground points can be described by the proportion of bins containing ground points [35]. This feature can be used to classify objects which are adjacent to ground.
- Roughness $F_{R}$
  Roughness can be determined by the variance in distances between the points and the fitting plane computed on its kernel size, namely the scale of a sphere containing nearest points. Smooth surface, such as roofs and facades, can be distinguished by this feature from other classes (e.g., cars, vegetations).
- Compactness $F_{C}$
  Compactness can be measured by the volume of the convex hull divided by the area for each cluster. The number of points in a cluster is defined here as the area. A small compactness will be obtained for erect or small size classes.
- Normal correlation $F_{N}$
  This feature can be measured by the correlation between normal vectors of cluster and the vertical direction of the horizontal plane, which has shown a better performance for regular classes compared with other classes.

**Figure 4:**
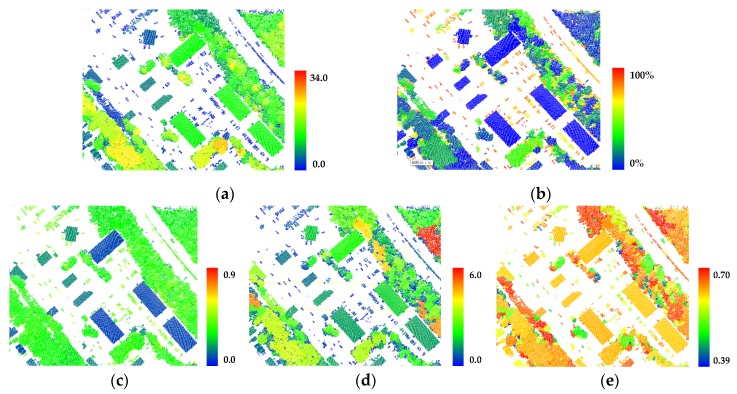
Cluster-based features: (**a**) height $F_{H}$; (**b**) distribution of ground points $F_{G}$; (**c**) roughness $F_{R}$; (**d**) compactness $F_{C}$; (**e**) normal correlation $F_{N}$.

All above cluster-based features have been proven effective in distinguishing one or more classes from others. As shown in Figure 4, each feature’s capacity is distinguished with different color-coded values. 

### 2.2. The LCS-CRF Model 

To conveniently describe the semantic classification problems, we first establish the notations and definitions used throughout the paper. Consider the input ALS point cloud $V=\left\{v_{1},v_{2},\cdots ,v_{N}\right\}$, where $v_{i}$ (i∈V={1,2,⋯,N}) represents a 3D point corresponding to the vertices in a graphical model, and N is the total number of points. A labeled point cloud can be represented by vector y∈Ω, containing the labels $y_{i}$ for all points. $y_{i}$ takes its value from the label set L={1,2,⋯,l}, where l denotes the number of classes. Edges $e_{ij}\in E$ are used to model the relations between pairs of adjacent points $v_{1}$ and $v_{2}$. Then, an undirected graphical model with graph G(V,E) consisting of nodes V and E can be constructed. 

#### 2.2.1. Pairwise CRF Model

Pairwise CRF model is widely used in semantic classification [13,36,37] to model the spatial interaction in both the labels and observed values, which is of importance in semantic classification. It is a discriminative classification approach, which directly models the posterior probability of the label y conditioned on the observed data x [38,39]. No more than two kinds of cliques are defined in a pairwise CRF. With the Hammersley–Clifford theorem, the CRF model as a Gibbs distribution can be modeled by:

(6) $$P(y|x)=\frac{1}{Z(x)}\exp\left\{-\sum_{c\in CG}\phi c(yc|x)\right\},$$

where Z(x) is the partition function, $C_{G}$ the set of all the cliques, and ϕc(yc|x) the potential function defined over the clique c to model the relationship of the random variables. An assignment of all the random variables (i.e., a labeling) takes values from $\Omega :=L^{N}$. Based on the Bayesian maximum a posteriori rule, the most likely labeling y∗ is inferred based on the given observation, which can be described as:

(7) $$y^{*}=\arg\;\underset{y\in \Omega }{\max}P(y|x).$$

The semantic classification problem with pairwise CRF model is therefore equivalent to finding the minimization of the Gibbs energy function E(y|x), which can be described by the sum of the unary and pairwise potentials. As a special case of Equation (6), E(y|x) is formulated as:

(8) $$\begin{array}{lll} E(y|x) & = & -\log P(y|x)-\log Z(x)\\ & = & \sum_{i\in V}\phi_{i}(y_{i},x)+\sum_{(i,j)\in E}\phi_{ij}(y_{i},y_{j},x) \end{array},$$

where $\phi_{i}$ is the unary potential term, a proxy for the initial probability distribution across semantic classes, and $\phi_{ij}$ is the pairwise potential term to keep smoothness and consistency between predictions. 

#### 2.2.2. LCS-CRF Model

Compared with pairwise CRF, richer statistics of point cloud can be captured by LCS-CRF. The problem of misclassification among different classes can be efficiently addressed by encoding higher-order semantics information, which can be employed in CRF model to improve the semantic classification performance. In our work, the potential functions are divided in three parts (i.e., unary, pairwise, and higher-order potentials) based on various cliques:

(9) $$\underset{y}{\max}P(y|x)\Leftrightarrow \underset{x}{\min}E(y|x)=\sum_{i\in V}\phi_{i}(y_{i},x)+\sum_{(i,j)\in E}\phi_{ij}(y_{i},y_{j},x)+\sum_{c\in C}\phi_{c}(y_{c},x),$$

where C represents the set of higher-order cliques, and $\phi_{c}$ are the higher-order potentials defined over cliques.

**Figure 5:**
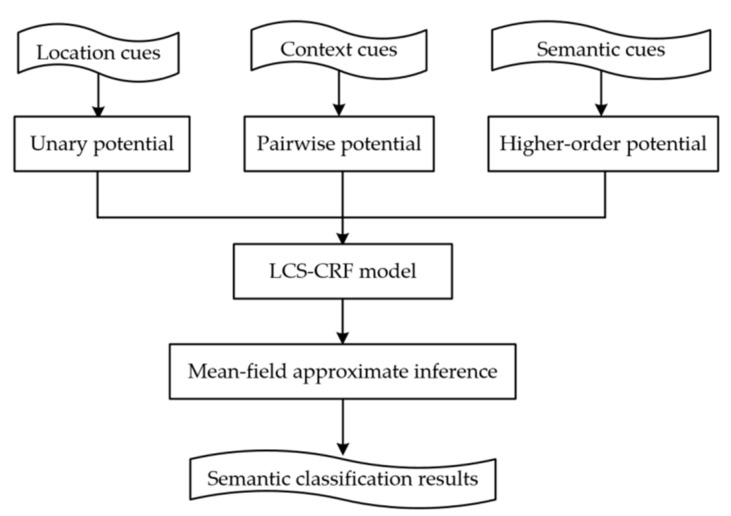
Flowchart of the LCS-CRF-based semantic classification in this study.

Then, the mean-field approximate inference algorithm is employed to optimize the energy function to obtain the final labels. Specifically, the location, context, and semantics are congregated in a higher-order CRF model, and the flowchart of the LCS-CRF-based semantic classification implemented in our study is shown in Figure 5.

### 2.3. LCS-CRF Energies 

#### 2.3.1. Point-based Features for Unary Potentials

The location information of point $v_{i}$ and its optimal neighbors are used to determine the point-based feature vectors, by which the unary potentials $\phi_{i}$ linking the point to the class labels determines the most probable label for a single point. The unary potentials $\phi_{i}$ can be defined by a discriminative classifier with a probabilistic output [40]. 

An ensemble learning method, RF classifier is employed to produce the soft labeling results for the unary potentials. RF classifier, constructing a multitude of decision trees during training and integrating the class probabilities of the individual trees at a testing stage, has shown a superior performance based on its robustness, high accuracy, and feasibility for ALS data [9]. In the implementation, each decision tree casts a vote for the most likely class. If the number of votes casts for a class l is $N_{l}$, the unary potential is defined by

(10) $$\phi_{i}{{}_{,}}_{RF}(y_{i}=l,x)=-\ln(N_{l}/N_{t}),$$

where $N_{l}$ is the total number of decision trees. Based on the point-based features, the location cues are directly used to discriminate the ALS points by the class membership probabilities.

#### 2.3.2. Weighted Potts Model

The pairwise potential $\phi_{ij}$ incorporates the contextual cues based on the spatial smoothing dependence principle. Based on the prior spatial knowledge, neighboring points are expected to take the same label. The weighted Potts model has been shown to work well for semantic classification in many previous studies [41,42]. Herein, the pairwise potential takes the form of:

(11) $$\phi_{ij}(y_{i},y_{j},x)=\mu (y_{i},y_{j})\left[w_{1}\exp(\frac{||p_{i}-p_{j}|{|}^{2}}{2\theta_{1}^{2}})+w_{2}\exp(-\frac{||x_{i}-x_{j}|{|}^{2}}{2\theta_{2}^{2}}-\frac{||p_{i}-p_{j}|{|}^{2}}{2\theta_{3}^{2}})\right],$$

where x and p represent the observed values and 3D coordinates. The label compatibility function μ(⋅), the weights of the spatial kernel and bilateral kernel $w_{1}$ and $w_{2}$, and the parameters of Gaussian kernels $\theta_{1}$, $\theta_{2}$, and $\theta_{3}$ are learned on the training set with the implementation provided in Reference [43]. 

Based on the spatial relationship, contextual relations between classes can be modeled and weighting factors are defined depending on how likely two classes occur near each other. 

#### 2.3.3. Higher-Order Potentials

**Figure 6:**
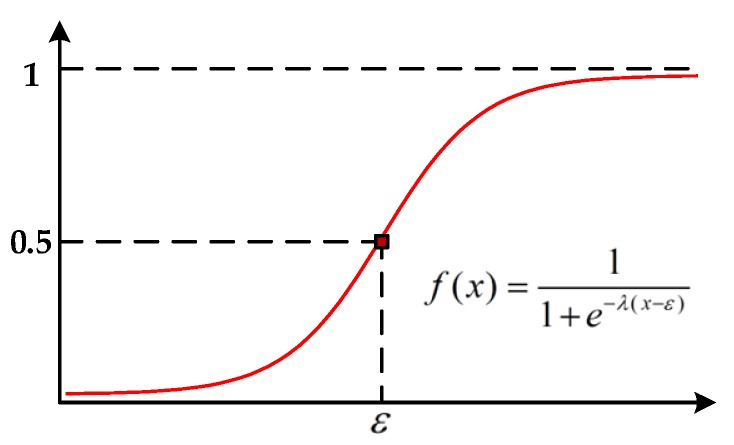
The Sigmoid function with scaling parameter λ and translation parameter ε.

Higher-order potentials are incorporated in a CRF model to capture richer perception between features and classes with semantics cues. In our work, the higher-order potentials are directly modeled by the cluster-based features with a sigmoid function. The sigmoid function is usually used as the activation function in many classification methods [44,45,46], which can be seen in Figure 6. 

Before computing the higher-order energy of CRF defined in (9), the cluster-based features are normalized in [0,1] to balance the perception between features and classes. Furthermore, because some features are only discriminated and beneficial for specified classes, the perception of all of the cluster-based features with regard to the labels on two test datasets, described in Section 3.1, can be summarized in Table 2, respectively. To simplify the description, the perception between a normalized feature f and each label y, R[•], can be modeled by:

(12) $$R[f,y]=\left\{ \begin{array}{ll} \frac{1}{1+e^{-\lambda (f-\varepsilon )}} & \;if\;“\propto ”\\ 1-\frac{1}{1+e^{-\lambda (f-\varepsilon )}} & \;if\;“-\propto ”\\ \;0 & \;if\;“/” \end{array}\right.,$$

where λ is the scale parameter, and ε the translation parameter. 

Specifically, some semantic rules are defined to adjust the higher-order potentials. Discriminative thresholds $\tau_{H}$ and $\tau_{G}$ for $F_{H}$ and $F_{G}$, respectively, can be used to classify buildings and vehicles. Buildings and facades have a lower value in $F_{R}$, which must be smaller than a threshold $\tau_{R}$. The values of $\tau_{H}$, $\tau_{G}$, and $\tau_{R}$ are semantically defined based on common knowledge, and are generally suitable in all scenes. Then, the higher-order potentials are defined as:

(13) $$\begin{array}{lll} \phi_{c}(y_{c}=l,x) & = & -\ln(S(y_{c},x))\\ S(y_{c}=l,x) & = & \left\{ \begin{array}{cc} 0, & if\;against\;rules\\ \sum_{f_{c}\in N_{F}}R[f_{c},l], & otherwise \end{array},\right. \end{array}$$

where ${Ν}_{F}=[f_{H},f_{G},f_{R},f_{C},f_{N}]$ is the normalized set of cluster-based features. We consider that off-ground points in a cluster share the same higher-order potential. To reduce the complexity of inference, the higher-order potentials can be rewritten by class membership probabilities and turned into unary potentials [43]. The integrated unary potentials can be written as:

(14) $$\phi_{i}(y_{i}=l)=\left\{ \begin{array}{ll} -\ln(N_{l}/N_{t}), & if\;i\in G\\ -\ln[\zeta (\frac{N_{l}}{N_{t}})+(1-\zeta )\frac{S(y_{i}=l)}{\sum_{y\in L}S(y)}],\; & otherwise \end{array}\right.,$$

where ζ is a free parameter from 0 to 1, to compromise the location cues and semantics cues. 

### 2.4. Evaluation Metrics

For evaluation, we compare the derived semantic labeling to the ground truth on a per-point basis. The confusion matrix and five commonly used measures are employed. The evaluation metrics are represented by overall accuracy (OA), Kappa coefficient (KA), recall (*R*), precision (*P*), and *F*_1_-score. Generally, the number of examples per class is inhomogeneous in the test data, and then OA and KA are used to reflect the overall performance and the degree of consistency. Meanwhile, *R* represents a measure of completeness or quantity, and *P* represents a measure of exactness or quality. The *F*_1_-score is a compound metric which combines *P* and *R* with equal weights. Appendix C describes the formulas in detail.

## 3. Experimental Analysis

To evaluate the performance of the proposed LCS-CRF algorithm, experiments with two ALS data sets were performed on a Windows 10 64-bit, Intel Core i7-4790k 4.00GHz processor with 32 GB of RAM, using Python language. 

### 3.1. Study Areas

**Figure 7:**
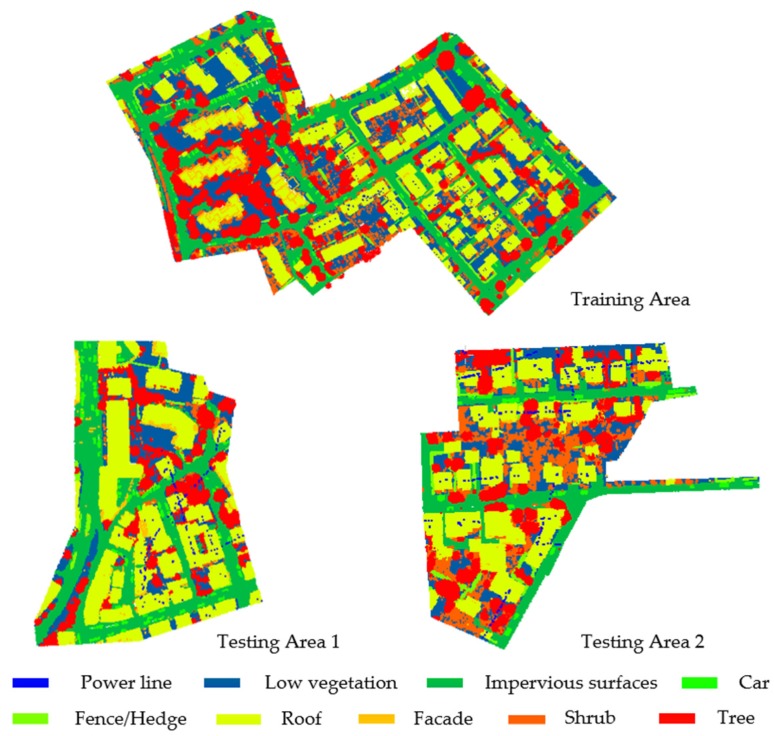
Vaihingen Dataset.

**Figure 8:**
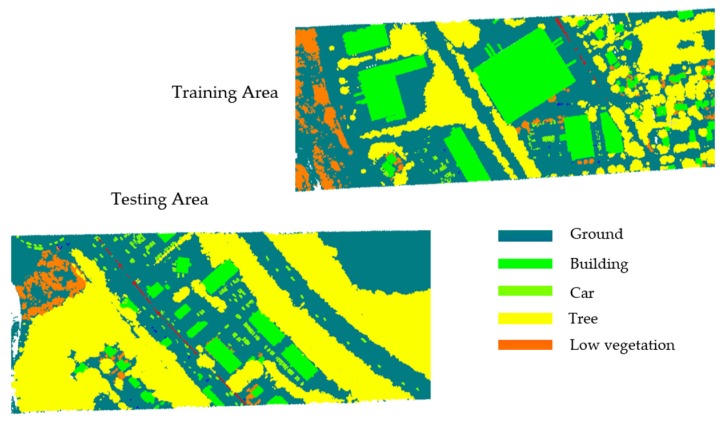
GML Dataset A.

Two labeled benchmark datasets, Vaihingen Dataset (Figure 7) and GML Dataset A (Figure 8), are employed to evaluate our methodology for ALS data of different characteristics. 

The Vaihingen Dataset is provided by the German Society for Photogrammetry, Remote Sensing and Geoinformation (DGPF) and was acquired with a Leica ALS50 system over Vaihingen, Germany, with an average point density of 4 points/m^2^. In the scope of the ISPRS Benchmark on 3D Semantic Labeling, a reference labeling was performed with respect to nine semantic classes, (namely, power line, low vegetation, impervious surfaces, car, fence/hedge, roof, facade, shrub, and tree). Thereby, each point in the data set is labeled accordingly [9]. For this dataset, containing about 1.166 M points in total, a split into a training scene (about 754 k points) and a test scene (about 412 k points) is provided. For each point, its XYZ-coordinates and intensity value are provided.

The GML Dataset A is provided by the Graphics & Media Lab, Moscow State University, and publicly available sources. This dataset has been acquired with an ALTM 2050 system (Optech Inc.) and contains about 2.077M labeled 3D points, whereby the reference labeling has been performed with respect to five semantic classes (namely, ground, building, car, tree, and low vegetation). For this dataset, a split into a training scene and a test scene is provided. For each point, its XYZ-coordinates are provided without intensity value.

### 3.2. Qualitative Comparison

In this section, we mainly focus on the analysis of three stages, i.e., ground points filtering, off-ground points clustering, and LCS-CRF performing.

**Figure 9:**
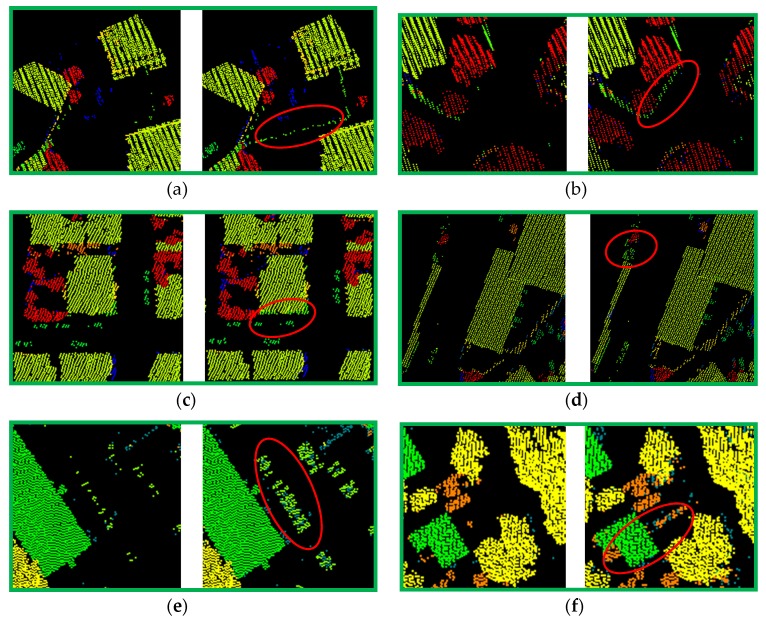

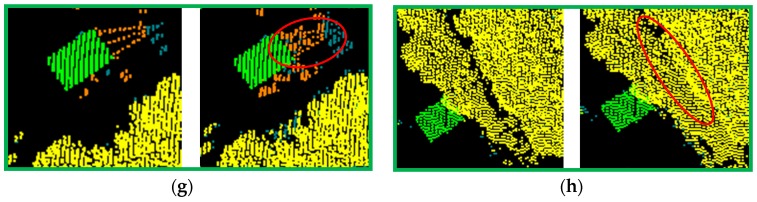
Comparison of CSF method with our off-ground points filtering method: (**a**–**d**) More *façade* points, *car* points, etc., can be integrated into off-ground points for Vaihingen Dataset; (**e**–**h**) more *car* points, *vegetation* points, etc., can be integrated into off-ground points for GML Dataset A.

To visually compare our proposed Algorithm 1 with the CSF method, some small parts with meaningful information are selected from Vaihingen Dataset and GML Dataset A, as shown in Figure 9. In Figure 9, each group (Figure 9a–h) presents the comparison of filtering results for off-ground point with CSF method and our proposed Algorithm 1. We can observe that some confusing object information, especially for small size objective, can be extracted from ground points set, which can be obtained from CSF method. Not only our method can extract off-ground points from ground point sets, but it can also enhance the reliability of higher-order potentials by eliminate the misclassification between off-ground and ground points. Yet, it has two shortcomings: (1) a fraction of ground points are filtered as off-ground points, which cause a coarse cluster-based classification result; and (2) different parameters should be explored for ALS data diversity. To overcome these shortcomings, we further consider the ground as one of the objectives classified in the calculation of higher-order potentials. Besides, sensitivity analysis for parameters is shown in Section 3.4.1. 

**Figure 10:**
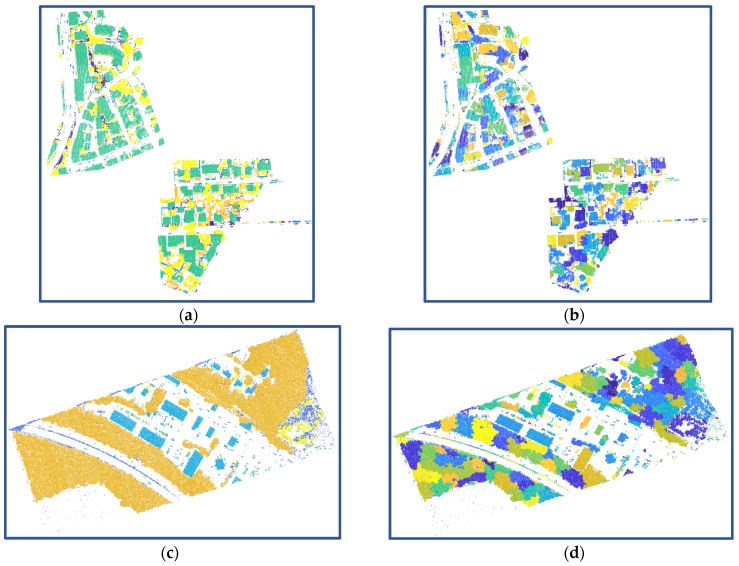
Filtered and clustering results of off-ground points from two ALS datasets: (**a**) off-ground points of Vaihingen Dataset obtained with Algorithm 1; (**b**) clusters of (a) obtained with Algorithm 2; (**c**) off-ground points of GML Dataset A obtained with Algorithm 1; (**d**) clusters of (c) obtained with Algorithm 2.

Compared with point-based features, the cluster-based features can provide new attributes, upon which semantics cues can be effectively employed. We define five cluster-based features for the derivation of higher-order potentials, which relate closely to the clustering results of off-ground points. Figure 10 presents the clustering results for the test data from Vaihingen Datasets and GML Dataset A, based on the off-ground points, which are extracted with Algorithm 1. As shown in Figure 10, class *roof* (green in Figure 10a)/*building* (blue in Figure 10c), which is far from ground with smooth surface; class *car* (cyan in Figure 10a and reseda in Figure 10c), which has a high correlation with ground; and class *tree* (yellow in Figure 10a)/*high vegetation* (orange in Figure 10c), which has a roughness surface, tend to be aggregated into single cluster, and we can make the utmost of semantics cues on these clusters. Due to the similarity of attributes for some different classes, mis-clusters, which means multiple classes contained in a cluster, also exist in the clustering results. Then, we employ the clustering result to define the higher-order potential in the LCS-CRF model, rather than the final semantic classification result. In the LCS-CRF model, we integrate the point-based features and cluster-based features, which show different attributes for each point and complement mutually. 

**Figure 11:**
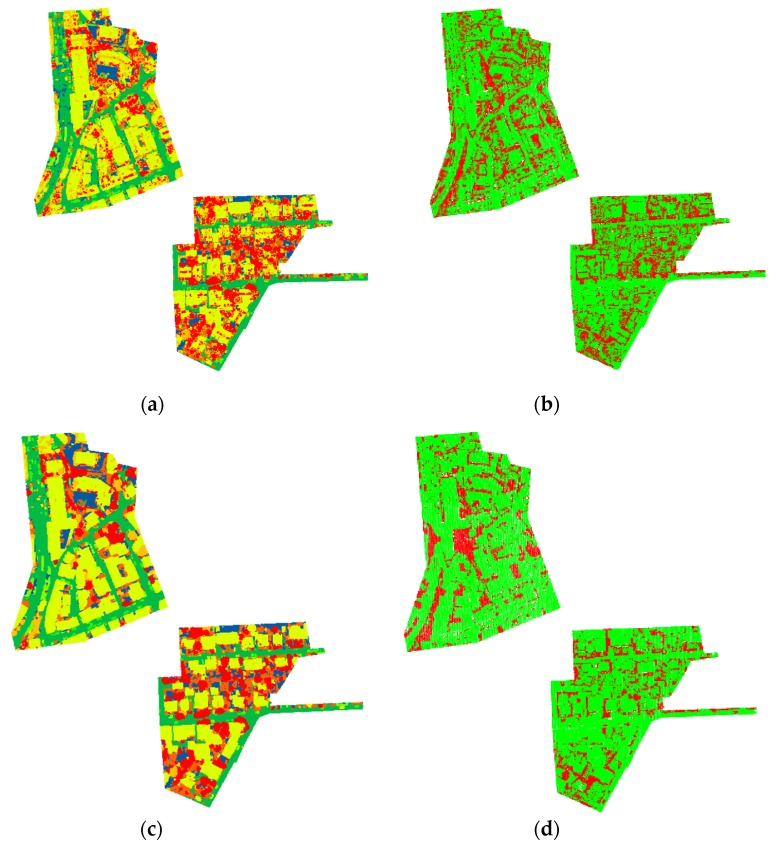

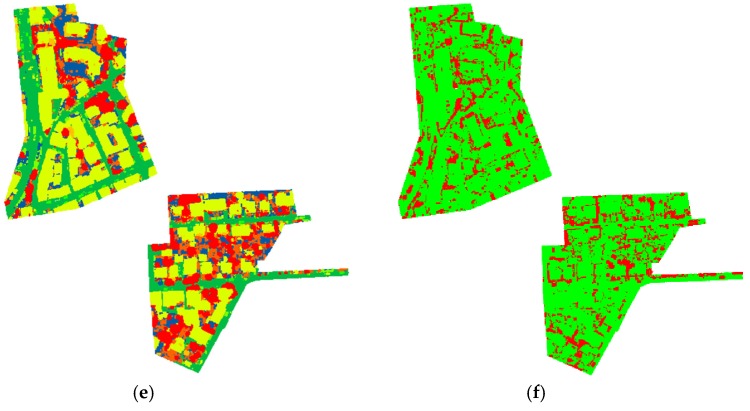
The semantic classification results and classification errors for the Vaihingen Dataset: (**a**,**b**) RF; (**c**,**d**) CRF; (**e**,**f**) LCS-CRF.

**Figure 12:**
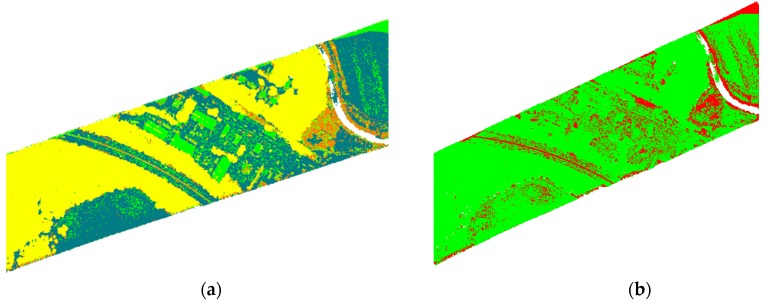

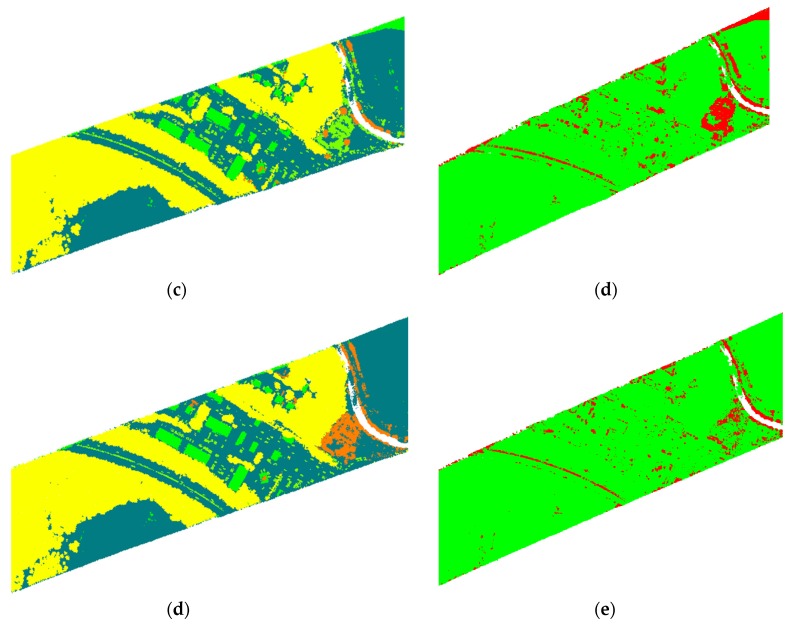
The semantic classification results and classification errors for the GML Dataset A: (**a**,**b**) RF; (**c**,**d**) CRF; (**e**,**f**) LCS-CRF.

To better evaluate the effectiveness of the LCS-CRF model, the qualitative results for three classification algorithms (i.e., RF, CRF, and LCS-CRF) of the two test datasets are, respectively, shown in Figure 11 and Figure 12. To learn the RF models, 400 trees are sufficient in our work. One thousand training samples for each class are randomly chosen from the reference ground-truth data of Vaihingen Dataset and GML Dataset A. The performance of RF in the case of limited training samples can be shown in Figure 11a,b and Figure 12a,b. The soft labeling results for each class, produced by RF, are considered as the unary term of CRF and LCS-CRF. 

As can be seen in Figure 11a,b, RF results in a discontinuous shape with lots of discrete points, due to the lack of consideration for the spatial contextual information. By considering the contextual information to alleviate the effect of noise, CRF can deliver a smoother classification map. Although the classification performance of a CRF model can be promoted dramatically by combining contextual information compared with RF method, their classification performance in keeping useful details are different. Due to the similarity between point-based features of different classes, e.g., ground and low vegetation, tree, and roof, etc., misclassified points are aggregated together, as shown in Figure 11d, which always directly affects the accurate interpretation of the various classes. It is a challenging task to accurately discriminate similar classes. However, on the whole, our proposed LCS-CRF model can achieve the semantic classification result with fewer misclassified regions and less salt and pepper classification noise by employing location-contextual-semantics cues. As shown in Figure 11e–f, the proposed model shows a competitive visual performance and can preserve useful detail information.

To verify the robustness of our method, another high-resolution ALS data of a different sensor is used to assess the performance of proposed method. Similarly, the semantic classification results of GML Dataset A obtained by three methods, i.e., RF, CRF, and LCS-CRF, are shown in Figure 12. Similar to the above test, CRF can deliver smoother results than RF and an improvement in the classification accuracy. Compared with RF model, CRF tends to greatly reduce the classification noise based on context cues. Then, some potentially useful details may also be eliminated. In this experiment, there is a slight difference of point-based features between the class car and low vegetation, which are easily confused. As shown in Figure 12c,d, an obvious misclassification has been presented. Most *low vegetation* points are classified as *car*, which limited the accuracies of the low vegetation and car. With the proposed LCS-CRF model, not only the location and context information are considered, but also the semantics to alleviate the misclassification effectively are fused. The visual results in Figure 12e,f, show an improvement for the *car* and *low vegetation* classification. 

It is observed that our proposed method outperforms RF and CRF. An improvement in the quantitative metrics will be analyzed in the next section, in which the quantitative performances of Vaihingen Dataset and GML Dataset A are also reported.

### 3.3. Quantitative Comparison 

In this section, the corresponding quantitative performances of Vaihingen Dataset and GML Dataset A are reported and analyzed. In accordance with Figure 11e,f and Figure 12e,f, our method can correctly label most of the test data. It can achieve a high OA of 83.1% and KA of 78.5% on the Vaihingen Dataset with eight categories of objects and a high OA of 94.3% and KA of 89.3% on the GML Dataset A with five categories of objects.

We classify semantic classification methods for the Vaihingen Dataset into two categories: traditional machine learning-based and deep learning-based. We compare our method with the result provided in Reference [26] and the submitted results with published papers provided by the ISPRS Semantic Labeling Benchmark. Reference [5,47,48] adopted the traditional machine learning classifiers to classify ALS point clouds, while Reference [49,50,51,52] leveraged deep learning for the semantic classification. For the sake of clarity and readability, the results achieved by each research group and our model (namely LCS-CRF) are listed for comparison in Table 3.

We perform experiments on another ALS dataset, i.e., GML Dataset A, to verify the effectiveness of our method. The LCS-CRF model ranks first in terms of the OA and $\bar{F_{1}}$ compared with other methods listed in the Table 4. 

### 3.4. Sensitivity Analysis for Parameters 

In our experiments, the LCS-CRF model obtained a good classification performance. However, there are so many parameters in the LCS-CRF model to be determined, which play an important role in the classification. These parameters distribute in three parts, i.e., Algorithm 1, Algorithm 2, and higher-order potentials.

#### 3.4.1. Parameters for Algorithm 1

**Figure 13:**
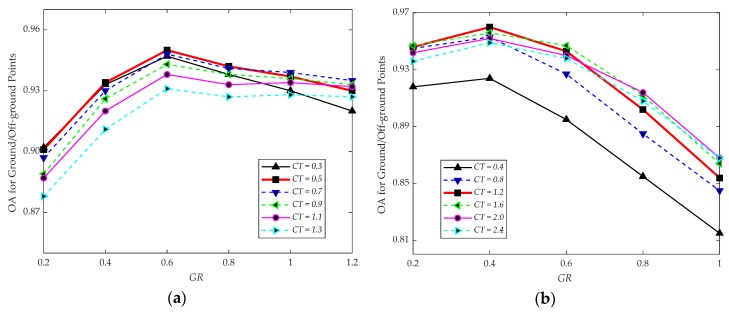
(**a**) Parameters analysis of CSF algorithm for Vaihingen Dataset; (**b**) Parameters analysis of CSF algorithm for GML Dataset A.

The implementation of CSF requires three essential parameters, including the GR to determine the number of particles, CT to select the distances between points and the simulated terrain, and MI to end the simulation process. To study the sensitivity of GR and CT for the CSF algorithm, MI is set to be 200, which is enough for our scene. GR varies from 0.2 to 1.2 and 0.2 to 1.0 for the test data of Vaihingen Dataset and GML Dataset A, respectively, with a step of 0.2. CT selected from 0.3 to 1.3 and 0.4 to 2.4 for the test data of Vaihingen Dataset and GML Dataset A, respectively, with a step of 0.4. Sensitivity analysis for parameters is presented in Figure 13. As can be observed, better results can be obtained, which are considered as the initial input of Algorithm 1, with GR equal to 0.6 and CT equal to 0.5 for Vaihingen Dataset, and GR equal to 0.4 and CT equal to 1.2 for GML Dataset A.

Although the ground points filtering accuracy can be 95.1% and 96.0% for Vaihingen Dataset and GML Dataset A, more detailed off-ground object information, especially objects with small size and low height, are essential for our scene to improve the semantic classification results. Then, we employ RANSAC for the ground points obtained by CSF to enrich the off-ground information with enough filtering accuracy. The property of RANSAC for each point is mostly determined by two thresholds, the maximum distance to distinctive initial inliers among current point’s neighbors and the minimum inlier ratio to determine whether the current point is an element of ground points set on the premise that the current point belongs to initial inliers.

**Figure 14:**
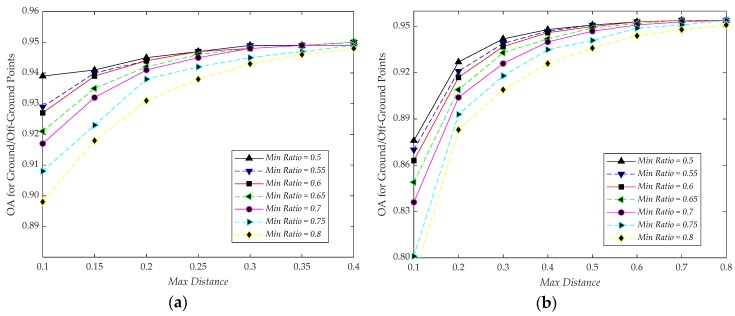
(**a**) Thresholds analysis for RANdom SAmple Consensus (RANSAC) algorithm on Vaihingen Dataset; (**b**) thresholds analysis for RANSAC algorithm on GML Dataset A.

To find an appropriate value of maximum distance and minimum inlier ratio, we test the procedure with maximum distance varying from 0.1 to 0.4 with a step of 0.05 and 0.1 to 0.8 with a step of 0.1 for the test data of Vaihingen Dataset and GML Dataset A, respectively. It is worth noting that 0.4 and 0.8 are not the cut-off values of maximum distance, only representing the variation tendency of OA for ground/off-ground points. The minimum inlier ratio varies from 0.5 to 0.8 with a step of 0.05 for the two test datasets. For evaluating the filtering results, we utilize OA for ground/off-ground points to analyze the OA. Analysis for these two parameters are shown in Figure 14. We can observe that the OA for ground/off-ground points converges to a certain value, due to the higher maximum distance and the lower minimum inlier ratio with a slight influence on the OA. To make the results more reliable, the observed results, which can show the details directly, parts of them as shown in Figure 9, are also considered to determine the values of these two parameters.

In order to obtain more details of off-ground object information and keep the OA, the parameters are utilized to perform Algorithm 1, which are listed in Table 5. These parameters are determined based on the experimental results (as shown in Figure 9 and Figure 14) and the properties of the input ALS point cloud.

#### 3.4.2. Parameters for Algorithm 2

Algorithm 2 is proposed to produce clusters of off-ground points, which can be used to extract discriminative cluster-based features. In the first step, two parameters, r and γ, are selected for the mean-shift algorithm, which are based on the prior knowledge about the expected point distribution for the scene we consider. Then, parameters k, $th_{d}$, and $th_{t}$, which were described in Section 2.1.3, are determined for the post-processing step. 

Herein, the performance of Algorithm 2 is mainly evaluated based on the intuitive result, and an experiment example has been shown in Figure 3. Then, we only provide the configuration of these parameters for Vaihingen Dataset and GML Dataset A, which is shown in Table 6.

#### 3.4.3. Parameters for Higher-Order Potentials

**Figure 15:**
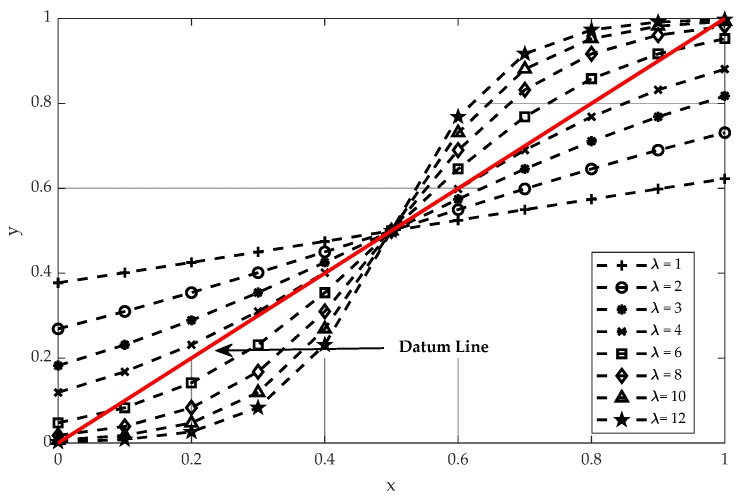
Sigmoid functions with different λ in the interval from 0 to 1.

In the LCS-CRF model, the higher-order potentials are derived with semantics cues based on a Sigmoid function. Two parameters are utilized to determine the formulation of Sigmoid function, and they are, respectively, denoted as λ and ε. Parameter λ mainly controls the scaling of Sigmoid function, while ε controls the translation. In this section, we also normalize the cluster-based features into [0,1], and then parameter ε is set as 0.5 to consist with the distribution of cluster-based features. The expression of Sigmoid function with different values of parameter λ is shown in Figure 15. The datum line is represented by a red straight line, which is treated as a reference to Sigmoid function. It means that the values of cluster-based features are directly used for the calculation of higher-order potentials. Different curves in the figure represent the projection values of cluster-based features through Sigmoid function with different λ. We employ Sigmoid function to enhance the discrimination of the cluster-based features to obtain a better classification result. However, there have been a few misjudgments in terms of cluster-based features, which are utilized to obtain the higher-order potential based on the regulations described in Section 2.3.3. Then, the corresponding analysis for parameter λ is given to test its effect in the LCS-CRF algorithm.

**Figure 16:**
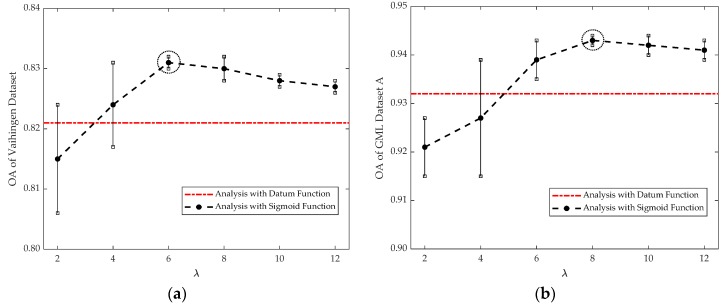
(**a**) Parameter λ analysis in Vaihingen Dataset; (**b**) Parameters λ analysis in GML Dataset A.

In order to study the sensitivity of the parameter λ for our method, other parameters are set to be constants. Experiments are conducted to analyze the effect of the parameter λ, which is varied from 2 to 12 with a step of 2 for Vaihingen Dataset and GML Dataset A. The sensitivity analysis for the parameter λ is presented in Figure 16. To make them more concise, we also compute the variation tendency of the OA under different settings of parameter λ, as shown in Figure 16a,b. The parameter λ shows obvious impact on the OA compared with employing the datum function, and the relative importance of the higher-order potential is increased as parameter λ increases.

We can observe that, the OA first increases as parameter λ increases since the semantic rules are properly utilized with Sigmoid functions to enhance the discrimination of cluster-based features. Then, the OA no longer increases at a certain value of parameter λ (i.e., around 6 for Vaihingen Dataset and around 8 for GML Dataset A), and even shows a slight decreasing trend, since the large varying degrees of cluster-based features can lead to the accumulation of noise from cluster-based features and cause misjudgments of clusters. The red dotted lines in Figure 16, serving as a reference, represents the classification results based on the higher-order potentials derived by datum function.

**Figure 17:**
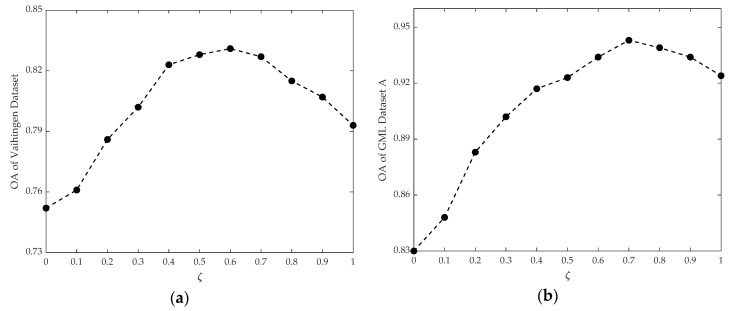
(**a**) The trends of the OA with parameter ζ in the Vaihingen Dataset; (**b**) the trends of the OA with parameter ζ in the GML Dataset A.

Another parameter, ζ, is also analyzed with Vaihingen Dataset and GML Dataset A, which mainly controls the effect of the higher-order potentials in the classification. As shown in the Figure 17, parameter ζ is selected from 0 to 1 with a step of 0.1, while other parameters are set to be constant values. The OA gradually increases in the beginning with the increase in parameter ζ, in which the semantic rules dominate the tendency compared with location information in the unary potential. After parameter ζ reaches up to a certain value (i.e., around 0.6 for Vaihingen Dataset and around 0.7 for GML Dataset A), the OA also shows a slight decreasing trend, since the unary potential become dominant with the increase in parameter ζ. When ζ equals to 1, the overall accuracies for Vaihingen Dataset and GML Dataset A reach 0.783 and 0.924, respectively, where the classification result is obtained by the CRF model. It is found that an obvious improvement of the classification results was shown in both test datasets by integrating higher-order potentials, compared with the results directly derived by CRF model.

## 4. Discussion

From Table 3, we can observe that the OA of LCS-CRF model performs the best among all of the traditional machine learning based method. As far as the eight specific classes are concerned, our method ranks first in the *imp_sur*, *car*, and *shrub* classes within the traditional machine learning-based methods, and its *P* surpass previous highest results with absolute advantages (+1.1%, +2.6%, and +6.1%). The RF model is mainly based on the point-based features, which are derived by the location cues of points, to perform semantic classification for ALS data. The CRF model integrates the location and contextual cues and shows a smoother result compared with the RF model (as shown in Figure 11). Obviously, the LCS-CRF model shows a superior result by incorporating location, context, and semantics cues into a higher CRF model. Especially for the *car* class, a great improvement of *P* is obtained by adding semantics cues. The class *low-veg*, with a higher *P*, mainly benefits from Algorithm 1. The OA of the LCS-CRF model ranks first among the traditional machine learning-based methods and third among the deep learning-based methods, with minor disadvantages (1.8% and 2.1% lower than the second and the first OA, respectively). Though some deep learning-based methods perform better than our method, the LCS-CRF model can also satisfy the general demand with less training costs.

In Table 4, the *P* of *car* class with LCS-CRF model surpass the results of RF and CRF model with +26% and +22.5%, which means that semantics cues play an important role in the semantic classification. We perform the methods *RF+LBP* and *RF+α-exp* by adding a regulation framework to smooth the semantic results derived by RF model. Though significant improvements are shown in *building*, *car*, and *low vegetation* classes compared with RF model, the OA of methods *RF+LBP* and *RF+α-exp* are still less than 90%. The *P* of *car* class for our method is superior to others, and plausible results are shown in *ground*, *building*, *tree*, and *low vegetation* classes, which validate our proposed method.

In comparison to other approaches, our method shows several strengths. We compare the results achieved with our methodology to the ones obtained by recent approaches. Similarly, Reference [5] proposed a hierarchical higher-order CRF framework, in which, spatial and context were integrated via a two-layer CRF. The Robust $P^{n}$ Potts model was utilized to build the higher-order potential in their first layer CRF. Their framework iterated and mutually propagated context to improve the classification results. The results, with their framework on the Vaihingen Dataset, have been described in Table 3 (LUH), which showed outstanding performance in $\bar{F_{1}}$ and revealed a rather high quality of the results in several classes. In contrast, our methodology extra integrates semantic cue in a higher-order CRF, which is a one-layer CRF with neither iteration nor propagation of context, and shows obvious increases in class *car* and OA by 5.8% and 1.5%, respectively. Currently, the only approach delivering semantic classification results of higher quality (with OA = 85.2% and $\bar{F_{1}}$ = 69.3%) for the Vaihingen Dataset is the one presented by Reference [52] that leverages deep learning for the semantic labeling of ALS point clouds. Yet, a multi-convolutional neural network (MCNN) was trained to automatically learn deep features of each point from the generated contextual images across multiple scales, which was time-consuming in training process and had relatively high requirements to hardware, while the proposed LCR-CRF framework only employs explicit point-based and cluster-based features. Comparable results can be observed in Table 3 with *P* in classes *imp_sur* (+0.1%), *car* (+8.8%), *façade* (-1.9%), and *shrub* (−0.5), and with the OA (−2.1%). Compared with [49] and [50], which also adopted deep learning for the semantic classification, the OA is, respectively, raised by 1.6% and 1.5% in our framework and *P* in several classes shows better performance, especially in class *car*. Due to the consideration of multi-scale neighborhoods, Reference [26] obtained an improved performance on the GML Dataset A by exploring contextual information across different scales in the, respectively, extracted features, while we obtain the optimal neighbors with the algorithm proposed in Reference [7] and integrate meaningful semantic cues. As shown in Table 4, our method increases the OA by 3.8% and the $\bar{F_{1}}$ by 11.7%, and three of the five classes’ *P* are improved. The methods *RF+LBP* and *RF+α-exp*, which was performed based on the methodology proposed in Reference [25], constructed graph models and employed structured regularization for spatially smoothing semantic labeling of point clouds. In our method, not only spatial information is utilized, but also context and semantic cues are integrated in a posterior probability model. In contrast with these two methods, our method better addresses some hard-to-retrieve classes, such as classes *car* and low *vegetation*, and increases OA by 8.3% and 6.5%, as observed in Table 4.

Experiment results suggest that the LCS-CRF model shows superior performance on the semantic classification for ALS data. However, there are still some misclassification in the results. For the Vaihingen Dataset, classes *fence* and *facade* are at a disadvantage due to their attributes, including the small cardinal number, sparsity, and similar characteristics with some other classes. A close-up visual inspection shows that the class *fence* is often classified as class *low_veg* or *shrub*, which causes adverse effects on the OA and $\bar{F_{1}}$. For the GML Dataset A, classes *building* and *car* produce lower precisions compared with classes *ground* and *tree*. Based on the visual inspection of test data, class *building* with small height shows similar attributes to classes *ground* and *car*, due to its planarity and clustering. Class *low vegetation* with smaller clusters is easily classified as *car*, which is very sensitive to the *P* of class *car* due to the extremely small size of class *car* compared with the whole test dataset.

As shown in Section 3.3, parameters in three parts, i.e., Algorithm 1, Algorithm 2, and higher-order potentials, are analyzed. Most parameter values are tested in a general interval based on the attributes of point clouds and common experience. Based on the hardware described in Section 3, it takes about 1.5 h to calibrate the parameters in the first and second parts both on the Vaihingen Dataset and GML Dataset A. The decision of parameters in the third part need a heavier time cost due to the large-scale ALS point clouds, and the time for each inference on the LCR-CRF model is about 1.2 h. Then, parallel computing is utilized to speed up the process to a great extent. Once the parameters are determined, automatic interpretation can be performed on large-scale ALS point clouds. In addition, it takes only about 0.5 h to train a CRF model on the Vaihingen Dataset in our work, while the training time in a deep learning framework takes about three to six days [54]. 

## 5. Conclusions

In this paper, we presented an LCS-CRF model for ALS data semantic classification. The main novelty of this framework consists of the integration of location, context, and semantics cues from irregularly distributed ALS points to semantically labeled point clouds in a higher-order CRF framework. The method processes in three main stages, i.e., (i) feature extraction; (ii) off-ground points extraction and clustering; and (iii) classification. A total of 34 point-based features from their locations and 5 cluster-based features from off-ground points’ clusters are extracted to form the feature space. To effectively employ the semantics cues, off-ground points extraction and clustering are performed for the cluster-based feature extraction. Based on the location and semantics cues, the unary potentials and higher-order potentials can be derived by the RF classifier and the sigmoid function. Then, the context information between neighbor points is integrated in a higher-order CRF as a pairwise potential to smooth the classification results. Therefore, the location, context, and semantics cues are, respectively, formulated in unary, pairwise, and higher-order potentials within the probabilistic LCS-CRF model to alleviate the misclassification. The experiments with two ALS point cloud data sets confirm the competitive semantic classification performance of the proposed method in both the qualitative and quantitative evaluations.

However, parameters with different values are sensitive to the classification results. In our future work, further improvements aim at preserving more potentially useful details to improve the results with fewer parameters. We also intend to investigate the potential of deep learning adapted to the ALS point cloud data. 

## Figures and Tables

**Table 1:** Three types of point-based features used in this work.

Type	Components
Geometric Features	H, ΔH, $\sigma_{H}$, r, D, $\sigma_{D}$, k1, $\sigma_{k1}$, k2, $\sigma_{k2}$, $C_{g}$, $\sigma_{C_{g}}$, $C_{m}$, $\sigma_{C_{m}}$, N, $\sigma_{N}$, C, $\sigma_{C}$, V
	$r_{2d}$, $D_{2d}$, $\sigma (D_{2d})$
Local Shape Features	L, P, S, O, A, E, $\sum_{Es}$, ΔC
	$\sum_{Es,2d}$, $R_{2d}$
Primitive Features	I, σ(I)

**Table:** (**a**)

Label\Feature	$F_{H}$	$F_{G}$	$F_{R}$	$F_{C}$	$F_{N}$
Low vegetation	−∝	−∝	−∝	∝	∝
Ground	−∝	∝	−∝	∝	∝
Car	−∝	∝	∝	∝	∝
Fence	−∝	∝	∝	−∝	∝
Roof	∝	−∝	−∝	∝	∝
Facade	∝	∝	−∝	−∝	−∝
Shrub	∝	∝	∝	∝	∝
Tree	∝	−∝	∝	∝	∝

**Table:** (**b**)

Label\Feature	$F_{H}$	$F_{G}$	$F_{R}$	$F_{C}$	$F_{N}$
Ground	−∝	∝	−∝	−∝	∝
Building	∝	−∝	−∝	−∝	∝
Car	−∝	∝	∝	−∝	∝
Low vegetation	−∝	∝	∝	∝	∝
High vegetation	∝	−∝	∝	∝	−∝

**Table 3:** Scores per class for each method and corresponding overall accuracy (OA) and $\bar{F_{1}}$ (%).

Methods	*P* (%)								OA	$\bar{F_{1}}$
	low_veg	imp_sur	car	fence	roof	facade	shrub	tree		
MSF [26]	67.5	82.7	35.7	14.1	86.3	39.9	32.2	69.9	68.1	52.6
HM_1 [47]	83.8	89.1	51.4	36.6	91.6	61.9	38.6	77.9	80.5	66.4
UM [48]	78.6	88.0	89.6	28.8	93.6	**66.5**	38.8	71.8	80.8	59.0
LUH [5]	83.0	**91.8**	86.4	**49.5**	**97.3**	52.4	34.1	**87.4**	81.6	**68.4**
RF	83.0	88.2	15.9	11.9	91.0	23.4	26.9	66.9	71.2	51.7
CRF	83.8	89.5	73.6	18.4	92.0	34.4	30.8	74.2	78.3	59.3
LCS-CRF	**84.9**	89.3	**92.2**	29.6	91.9	45.7	**44.9**	76.5	**83.1**	60.8
BIJ_W [49]	77.1	88.5	61.6	55.7	92.5	**85.5.**	39.2	80.1	81.5	60.3
RIT_1 [50]	88.0	89.6	70.1	66.5	95.2	51.4	33.4	86.0	81.6	63.3
Whu Y4 [51]	80.6	**90.4**	71.0	**73.0**	93.1	62.4	**55.2**	81.9	84.9	69.2
NANJ2 [52]	**90.0**	89.2	**83.4**	50.5	**95.7**	47.6	45.4	**88.3**	**85.2**	**69.3**

MSF: the method based on multi-scale features.

**Table 4:** Scores per class for each method and corresponding OA and $\bar{F_{1}}$ (%).

Methods	*P* (%)					OA	$\bar{F_{1}}$
	Ground	Building	Car	Tree	Low Vegetation		
MSF [26]	97.5	47.2	17.2	98.7	10.8	90.5	58.5
AMN [53]	74.8	7.9	32.6	**98.8**	**88.7**	-	-
RF+LBP	95.3	66.8	13.3	97.9	14.6	86.0	57.3
RF+α-exp	94.0	**69.9**	14.0	98.1	17.3	87.8	57.6
RF	**98.5**	22.8	7.3	98.4	6.8	84.4	48.3
CRF	96.6	42.8	10.8	97.1	19.9	92.4	57.1
LCS-CRF	96.8	59.6	**33.3**	97.2	30.8	**94.3**	**70.2**

AMN: Associative Markov Networks; LBP: Loopy Belief Propagation.

**Table 5:** The Parameters setting of Algorithm 1 for Vaihingen Dataset and GML Dataset A.

Parameters	*GR*	*CT*	*MI*	*Max Distance*	*Min Ratio*
**Vaihingen**	0.6	0.5	200	0.6	0.7
**GML A**	0.4	1.2	200	0.9	0.8

**Table 6:** The Parameter configuration of Algorithm 2 for Vaihingen Dataset and GML Dataset A.

Parameters	r	γ	k	$th_{d}\;$	$th_{t}$
**Vaihingen**	2	1	20	1.4	0.8
**GML A**	3	1	30	1.4	0.9

## Appendix A

Parameters Max Iterations (MI)*,* Classification Threshold (CT), and Grid Resolution (GR) are utilized for the CSF algorithm which have been analyzed to obtain a better initial result in Section 3. The number of neighbors for a query point is determined by parameter k. th1 and th2 are used to determine the inliers and calculate the inlier proportion thresholds of the RANSAC algorithm and inlier proportion.

**Algorithm 1.** CSF with RANSAC
**Input**: *ALS point cloud set*$P=\{p_{1},p_{2},\cdots ,p_{n}\}$. **Parameters**: MI, CT, GR, k, th1, th2 1: *Ground points set* {G}←ϕ, *Off-ground points set* {NG}←ϕ 2: *Derive the initial ground and off-ground points with CSF* {G} & {NG}←(P,MI,CT,GR) 3: **For** *i = 0 to size* {G} **do** 4: *Find k neighbors of i-th query point* $p_{i}\leftarrow (i,k,P)$ 5: *Inliers are determined with RANSAC algorithm* $\{IP_{i}\}\leftarrow th1$ 6: *Calculate the proportion of* $\left\{IP_{i}\right\}$ *to all neighbors* $ir_{i}\leftarrow (th1,k)$ 7: **If** $p_{i}\in \{IP_{i}\}\;\&\;ir_{i}>th2$ 8: Keep $p_{i}\in \{G\}$ 9: **Else then** 10: $\{NG\}⇐\{NG\}\cup p_{i}$ 11: **End For** Output: {G}, {NG}

## Appendix B

Parameters r and γ, scales for neighbor size and gaussian kernel, respectively, are utilized for the mean-shift algorithm which have been analyzed to obtain a better initial result in Section 3. The number of neighbors for a query cluster is determined by parameter k. $th_{d}$ and $th_{t}$ are the constrains for local connectivity and structure correlation.

**Algorithm 2.** Constrained mean-shift algorithm
**Input**: *ALS off-ground point set*{NG}**Parameters**: r, γ, k, $th_{d}$, $th_{t}$ 1: *Derive the initial clusters and cluster centers with mean-shift algorithm* $\left\{C\}\&\{C_{cen}\}\leftarrow (NG,r,\gamma \right)$ 2: **While** *true* **do** 3: **For** j = 1 *to size* {C} **do** 4: **If** $C_{j}\in \{C\}$ **then** 5: *Find neighbors for each cluster* $N_{j}\leftarrow (C_{cen},k)$ 6: *Compare* $C_{j}$ *with cluster* $n_{j}\in N_{j}$ 7: **If** local connectivity < $th_{d}$ *and* structure correlation < $th_{t}$ **then** 8: $C_{j}\leftarrow C_{j}\cup n_{j}$, $\{C\}\leftarrow \{C\}\backslash n_{j}$ 9: **End If** 10: **End If** 11: **End For** 12: *No merging happened.* 13: **End While** **Output**: *Final cluster set*{C}

## Appendix C

The OA, KA, *R*, *P*, and *F*_1_-score can be computed by the confusion matrix as follows:

(A1) $$\mathrm{OA}=\frac{1}{N}\sum_{i=1}^{m}x_{ii}$$

(A2) $$\mathrm{KA}=\frac{N\sum_{i=1}^{m}x_{ii}-\sum_{i=1}^{m}\left[(x_{ii}+\sum_{j\neq i}^{m}x_{ji})\times (x_{ii}+\sum_{j\neq i}^{m}x_{ij})\right]}{N^{2}-\sum_{i=1}^{m}\left[(x_{ii}+\sum_{j\neq i}^{m}x_{ji})\times (x_{ii}+\sum_{j\neq i}^{m}x_{ij})\right]}$$

(A3) $$P=\frac{x_{ii}}{x_{ii}+\sum_{j\neq i}^{m}x_{ji}}$$

(A4) $$R=\frac{x_{ii}}{x_{ii}+\sum_{j\neq i}^{m}x_{ij}}$$

(A5) $$F_{1}=2\cdot \frac{P\cdot R}{P+R}$$

where $x_{ij}$ represents the element of confusion matrix on ith row and jth column.
